# Antimycobacterial Effect of Selenium Nanoparticles on *Mycobacterium tuberculosis*

**DOI:** 10.3389/fmicb.2020.00800

**Published:** 2020-04-28

**Authors:** Hector Estevez, Ainhoa Palacios, David Gil, Juan Anguita, Maria Vallet-Regi, Blanca González, Rafael Prados-Rosales, Jose L. Luque-Garcia

**Affiliations:** ^1^Department of Analytical Chemistry, Faculty of Chemistry, Complutense University of Madrid, Madrid, Spain; ^2^Inflammation and Macrophage Plasticity Lab, CIC bioGUNE, Derio, Spain; ^3^Electron Microscopy Platform, CIC bioGUNE, Derio, Spain; ^4^Ikerbasque, Basque Foundation for Science, Bilbao, Spain; ^5^Department of Chemistry in Pharmaceutical Sciences, Faculty of Pharmacy, Instituto de Investigación Sanitaria Hospital 12 de Octubre (imas12), Complutense University of Madrid, Madrid, Spain; ^6^Centro de Investigación Biomédica en Red de Bioingeniería, Biomateriales y Nanomedicina (CIBER-BBN), Madrid, Spain; ^7^Department of Preventive Medicine and Public Health and Microbiology, Faculty of Medicine, Autonomous University of Madrid, Madrid, Spain

**Keywords:** selenium nanoparticles, mycobacterium tuberculosis, antimycobacterial effect, *smegmatis*, cell wall damaging agents

## Abstract

Tuberculosis (TB) remains the leading cause of death from a single infection agent worldwide. In recent years, the occurrence of TB cases caused by drug-resistant strains has spread, and is expected to continue to grow. Therefore, the development of new alternative treatments to the use of antibiotics is highly important. In that sense, nanotechnology can play a very relevant role, due to the unique characteristics of nanoparticles. In fact, different types of nanoparticles have already been evaluated both as potential bactericides and as efficient drug delivery vehicles. In this work, the use of selenium nanoparticles (SeNPs) has been evaluated to inhibit the growth of two types of mycobacteria: *Mycobacterium smegmatis* (*Msm*) and *Mycobacterium tuberculosis* (*Mtb*). The results showed that SeNPs are able to inhibit the growth of both types of mycobacteria by damaging their cell envelope integrity. These results open a new opportunity for the use of this type of nanoparticles as antimycobacterial agents by themselves, or for the development of novel nanosystems that combine the action of these nanoparticles with other drugs.

## Introduction

Tuberculosis (TB) is a chronic infectious disease transmitted aerially through droplets expelled by an infected person. The disease is caused by the slow-growing tubercle bacillus *Mycobacterium tuberculosis* (*Mtb*). TB is the leading cause of death due to infection worldwide, with approximately 1.4 million deceased in 2018 (World Health Organization [WHO], 2019) and estimates at 75 million people dying from TB over the next 35 years. In 2018 alone, new TB cases were estimated to be 10.4 million worldwide (World Health Organization [WHO], 2019). Currently, TB treatment requires a 6-month regimen of four first-line drugs, which are ineffective at treating infection with multidrug-resistant (MDR) *Mtb* strains. These strains are rapidly spreading worldwide (more than 480,000 cases reported last year according to the TB alliance) and could cost $16 trillion over the next 35 years. Since microbial resistance to antibiotics increases, there is a call for non-antibiotic therapies that can fill the gaps for bactericidal purposes where antibiotics fail (Chandra Mohana et al., 2018). In the recent years, the combination of nanotechnology and biomedicine has proven to be promising for multiple purposes, including in bactericide applications (Jayawardana et al., 2015; Reshma et al., 2017; Vallet-Regí et al., 2019).

Nanoparticles are highly promising as antimicrobials, complementary to antibiotics, as they act by influencing the bacterial cell wall by direct contact without the need to be endocytosed (Herman and Herman, 2014; Wang et al., 2017). Their small size implies a greater surface-area-to-volume ratio than bulk material. Also, they may have optical or even magnetic properties (Baranwal et al., 2018). There is a great variety of nanoparticles in use as antimicrobials, such as metal, metal oxide or organic nanoparticles, involving multiple modes of action (Hoseinzadeh et al., 2017; Reshma et al., 2017; Khan et al., 2020). However, nanoparticles affect bacteria in two main lethal pathways, which can occur simultaneously: disruption of membrane potential and integrity, or production of reactive oxygen species (ROS) (Beyth et al., 2015).

Selenium nanoparticles (SeNPs) have already been suggested for multiple biomedical applications due to their antioxidant properties and differential behavior in comparison to other selenospecies (Estevez et al., 2014). Also, SeNPs have been shown to have antimicrobial activity against different types of bacteria (Huang et al., 2019). However, SeNPs have never been proven before to have an antimicrobial effect on *Mtb*.

Therefore, in this work, the mycobactericidal capacity of SeNPs has been evaluated on two types of mycobacteria, the fast-growing *Mycobacterium smegmatis* (*Msm*) and the slow-growing *Mtb*, finding an effective inhibition in the mycobacterial growth in a dose-dependent manner. A structural analysis of the mycobacteria subjected to the presence of the SeNPs has been performed by means of electron microscopy to analyze their effect on the cell envelope, showing that SeNPs interact directly with the mycobacteria membrane of *Mtb* and *Msm*, compromising their integrity and inducing extrusion of cytoplasmic material.

## Materials and Methods

### Chemicals

Reagents for the synthesis of SeNPs: chitosan, bovine serum albumin, sodium selenite, ascorbic, and acetic acid were purchased from Sigma-Aldrich. Reagents for electron microscopy: glutaraldehyde, cacodylate and formaldehyde were purchased from Sigma. Uranyl formate was purchased from Electron Microscopy Sciences (Hatfield, PA, United States).

### Synthesis of SeNPs

Chitosan-stabilized SeNPs (Ch-SeNPs) were synthesized following the procedure described by Bai et al. (2008). Briefly, 10 mL of an aqueous chitosan polysaccharide solution (0.5% w/v) were mixed with 7.5 mL of ascorbic acid 0.23 M and 5 mL of acetic acid 2.4 M. Then, 0.25 mL of sodium selenite 0.51 M were slowly added to the previous solution. SeNPs formation was recognized by observing the change of the solution from colorless to red as the reaction progressed. After the synthesis, the colloidal suspension was diluted to 50 mL with distilled water, resulting in final concentrations of 200 mg L^–1^ of Se and 0.1% of chitosan. Finally, the colloidal suspension was dialyzed for 2 h at room temperature in a ratio of 10 mL against 2 L of distilled water and using a 12 kDa of MWCO membrane.

For control experiments, BSA-SeNPs were also prepared following the same procedure but using an aqueous bovine serum albumin solution (0.5% w/v) instead of the chitosan solution.

### Analytical Characterization of the Synthesized SeNPs

Transmission electron microscopy (TEM) was performed using a JEOL JEM 1400 instrument operated at 120 kV equipped with a CCD camera (KeenView Camera) and an energy dispersive X-ray spectroscopy (EDS) analyzer. Sample preparation was performed by placing one or two drops of the SeNPs colloidal suspension onto carbon-coated copper grids.

Electrophoretic mobility measurements of the materials suspended in water were used to calculate the zeta potential (ζ) values of the nanoparticles. Measurements were performed in a Zetasizer Nano ZS (Malvern Instruments Ltd., United Kingdom) equipped with a 633 nm “red” laser. The hydrodynamic size of nanoparticles was measured by dynamic light scattering (DLS) with the same Malvern instrument.

### Bacterial Strains and Culture Conditions

*Mycobacterium smegmatis* mc^2^155 (*Msm*) and *Mycobacterium tuberculosis* H37Rv (*Mtb*) strains (obtained from the ATCC) were grown in Middlebrook 7H9 medium supplemented with 10% (v/v) OADC supplement (NaCl 8.5 g/L, BSA fraction V 50 g/L, dextrose 20 g/L, 5% (v/v) oleic acid solution 1%, 40 mg/L catalase), 0.5% (v/v) glycerol and 0.05% Tyloxapol (v/v; Sigma). Cultures were grown at 37°C in static standing 25 cm^2^ flasks with vented caps. *Msm*-Lux was generated by transforming *Msm* with the plasmid construct pMV306hsp + Lux (which contains the entire bacterial Lux operon cloned in a mycobacterial integrative expression vector) (Andreu et al., 2010).

### Minimum Inhibitory Concentration (MIC) Assay

Minimum inhibitory concentration (MIC) assay was carried out in 96-well plates diluting exponentially growing *Msm* or *Mtb* at an initial density of 1 × 10^5^ bacteria/mL in the presence of two-fold serial dilutions of Ch-SeNPs or BSA-SeNPs starting at 50 μg/mL and including one control sample with no NPs. Plates were incubated at 37°C for 7 days (*Msm*) or for 20 days (*Mtb*). Bacterial growth was monitored every day for *Msm* and every week for *Mtb* and was determined by measuring optical density at 570 nm. The assay was performed in triplicate. MIC values were selected as the minimum concentration able to suppress mycobacterial growth. Alternatively, *Msm*-lux was submitted to MIC assay and cell viability was assessed by measuring relative luminescence units (RLUs) at day 2. Correlation between colony forming units (CFUs) was performed according to a previously performed *in vitro* RLUs vs. CFUs curve.

### Transmission Electron Microscopy (TEM) (Negative Staining)

*Mycobacterium smegmatis* cells were fixed with 2% glutaraldehyde in 0.1 M cacodylate at room temperature for 2 h, and then incubated overnight in 4% formaldehyde, 1% glutaraldehyde, and 0.1% PBS. For negative staining of the samples, a drop of the fixed bacterial suspension was applied directly onto a glow-discharged EM grid (QUANTIFOIL. Formvar/Carbon. Cu 400 mesh grids). The sample was allowed to be adsorbed and then blotted with filter paper (Whatman grade No. 1). The grid was washed by touching the surface with two consecutive drops of 0.75% (w/v) uranyl formate, blotting each time, and stained for 1 min with one more drop of the same staining agent. Negative stained samples were examined in a JEOL JEM-1230 (accelerating voltage 100 kV) electron microscope, and images were recorded with a CCD camera ORIUS SC100 (4 × 2.7 k pixel).

### Cryo-Electron Microscopy (Cryo-EM)

*Mycobacterium smegmatis* and *Mtb* cultures were fixed as above prior to vitrification. Grids were prepared following standard procedures and observed at liquid nitrogen temperatures in a JEM-2200FS/CR transmission electron microscope (JEOL Europe, Croissy-sur-Seine, France) operated at 200 kV. An in-column omega energy filter helped to record images with improved signal/noise ratio by zero-loss filtering. The energy selecting slit width was set at 9 eV. Digital images were recorded on an UltraScan4000 CCD camera under low-dose conditions at a magnification of 55,058 obtaining a final pixel size of 2.7 Å/pixel.

## Results

### Synthesis and Characterization of SeNPs

Selenium nanoparticles were prepared via a redox system in the presence of a biomacromolecule as soft template to control the nucleation and growth of the inorganic nanoselenium. The chemical reduction of selenite with ascorbic acid in the presence of a polysaccharide such as chitosan as stabilizer and capping agent afforded red elemental selenium in colloidal state (Zhang et al., 2010). Based on our previous work, 0.1% of chitosan concentration was selected to prepare the SeNPs (Estevez et al., 2014). Under these conditions, the nanoparticles proved to be colloidally stable. Two months after their synthesis, no flocculated material was present, DLS measurements showed no displacement of the hydrodynamic size distribution and no significant differences in the shape or size of the SeNPs were observed by TEM.

**FIGURE 1:**
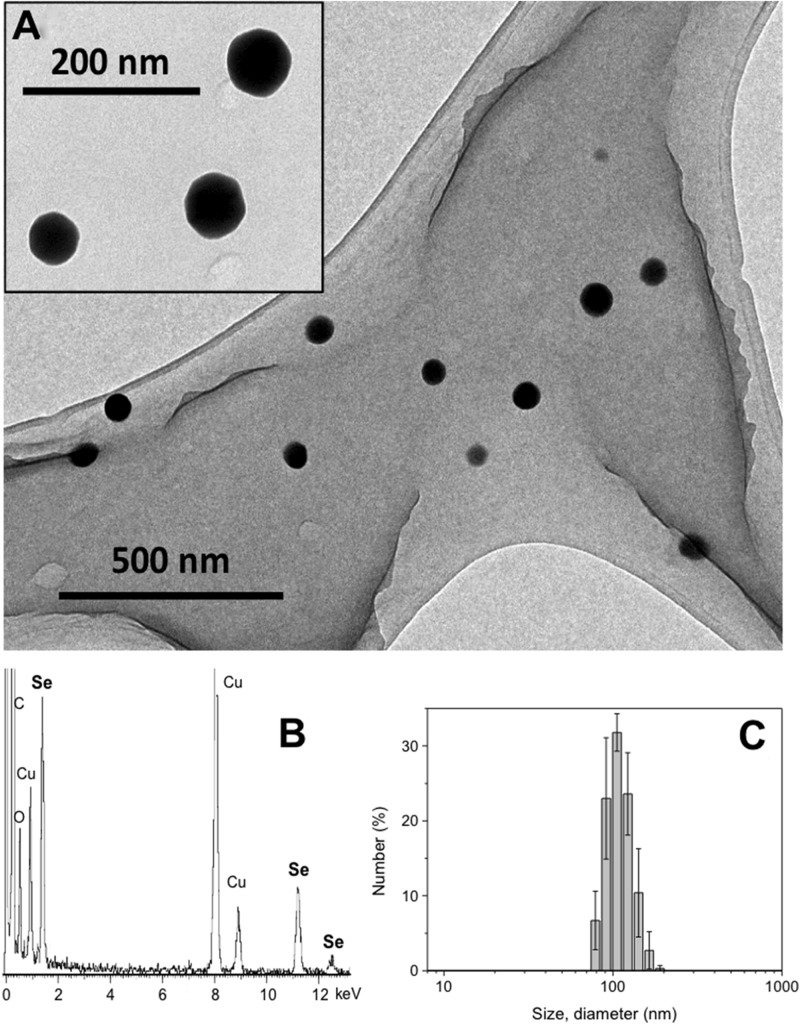
Characterization of the synthesized SeNPs. **(A)** TEM images of a 200 mg L^–1^ suspension of Ch-SeNPs. **(B)** EDS spectrum of Ch-SeNPs. **(C)** Hydrodynamic size distribution of the Ch-SeNPs in aqueous colloidal suspension measured by dynamic light scattering.

Transmission electron microscopy images show well dispersed Ch-SeNPs that exhibit spherical morphology and homogeneous sizes of around 60–80 nm (Figure 1A). The energy dispersive spectroscopy analysis (Figure 1B) indicates a composition in selenium for the Ch-SeNPs, also being observed in the spectrum the signals for C and O from chitosan and the carbon coated copper grid.

The hydrodynamic size distribution of the Ch-SeNPs measured in the aqueous colloidal suspension (Figure 1C) is monomodal and reasonably narrow, with a maximum centered at 105.7 ± 2.5 nm of diameter, which is in concordance with the smaller size of the inorganic nanoparticle determined in the TEM study. The ζ-potential of the Ch-SeNPs in water is in the zone of colloidal stability with a value of +66.6 ± 4.7 mV. This highly positive value is due to the surface stabilization with chitosan, which possesses a equilibrium for the protonation of the amino groups in water. Therefore, chitosan allows the SeNPs to form stable colloidal suspensions due to both electrostatic as well as steric stabilization.

### Antimycobacterial Activity of SeNPs

**FIGURE 2:**
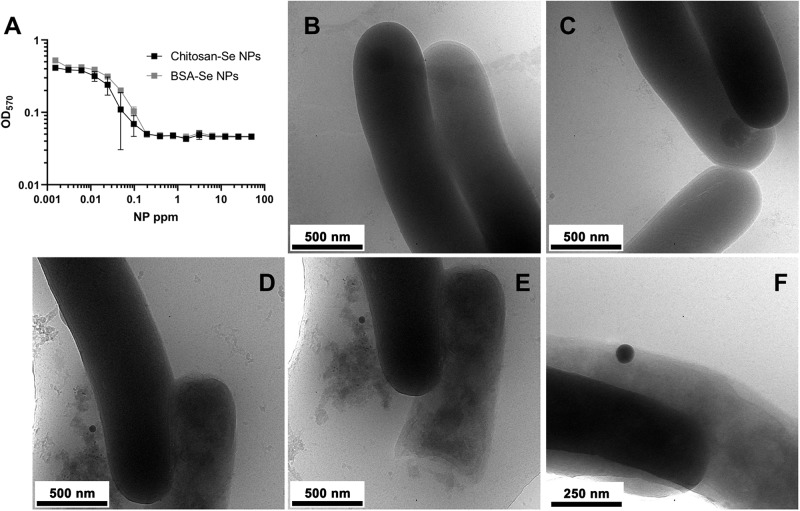
Antimicrobial activity of SeNPs against *Mycobacterium tuberculosis*. **(A)** MIC assay testing the antibacterial activity of Ch-SeNPs and BSA-SeNPs after 20 days of treatment. **(B–E)** Ultrastructural analysis by cryo-EM of untreated *Mtb* cells **(B,C)** or *Mtb* cells treated with an inhibitory concentration (0.195 μg/mL) of SeNPs **(D–F)**.

We evaluated the ability of SeNPs to inhibit the growth of two different species of mycobacteria: the fast-growing *Msm* and the slow-growing *Mtb*. SeNPs were found to be effective in inhibiting mycobacterial growth in a dose-dependent manner, showing MIC values of 0.400 μg/mL for *Msm* (Supplementary Figure S1A) and 0.195 μg/mL for *Mtb* (Figure 2A). To determine whether this inhibition was concomitant to bacterial cell death we generated a luciferase-expressing reporter strain of *Msm* (Msm-Lux) (Andreu et al., 2010). Correlation between relative fluorescence units (RLUs) and CFUs was previously determined (Supplementary Figure S2A) and used to calculate viable bacteria after treatment of *Msm*-Lux with SeNPs (Supplementary Figure S2B). We observed a reduction in CFUs as concentration of NPs increased, indicating the bactericidal effect of SeNPs. By this method MIC was similar to that of previously determined by measuring optical density.

To gain insight into the nature of the inhibitory effect of SeNPs, we performed ultrastructural analysis of *Msm* submitted to 0.400 μg/mL of SeNPs. Transmission electron micrographs of negative stained samples suggested that *Msm* exposed to SeNPs manifested reduced integrity of cell envelope leading to extrusion of cytoplasmic material, relative to untreated *Msm* (Supplementary Figures S1B,C). To capture the antimycobacterial effect of SeNPs in a close-to-native state, treated (0.195 μg/mL) and untreated *Msm* and *Mtb* cells were submitted to cryo-EM. This technique revealed more in detail the membrane damage, both in *Msm* (Supplementary Figures S1D,E) and *Mtb* (Figures 2B–F). In addition, SeNPs could be spotted in direct contact with the mycobacterial cell wall, suggesting a direct connection between the effect of SeNPs and the reduction of the integrity of the mycobacterial cell envelope.

## Discussion

The rapid spread of antibiotic-resistant *Mtb* strains makes it necessary to search for alternative treatments. In this sense, the use of nanoparticles with mycobactericidal potential could be especially interesting, since nanoparticles have a high surface area, which means that they contain a high number of active sites to be able to interact with biological entities and, on the other hand, they have a high capacity to penetrate cells and tissues (Lu et al. 2018; Singh et al., 2015).

Previous studies have used different types of nanoparticles, either as drug carriers (Lu et al., 2018) or as bactericidal agents themselves against TB. In the latter case, the mycobactericidal potential of different metallic nanoparticles such as Ag (Selim et al., 2018), AgO, AgZnO (Jafari et al., 2017) or Ga (Choi et al., 2017) nanoparticles, has been evaluated.

Among the different nanoparticles that are being used in biomedicine, Se nanoparticles are especially interesting because of their low toxicity. While Se has a narrow therapeutic window and the toxicity margins are very delicate, SeNPs possess remarkably reduced toxicity (Khurana et al., 2019) and it has been proposed as a therapeutic agent for different applications without significant side effects (Hosnedlova et al., 2018). As a matter of fact, it has been demonstrated that SeNPs are less toxic than other inorganic and organic selenospecies (Estevez et al., 2014), showing unique properties such as their capacity for inducing cell cycle arrest (Lopez-Heras et al., 2014) without inducing a significant degree of apoptosis (Estevez et al., 2014). In addition, SeNPs have also been used as a bactericidal agent against *Staphylococcus aureus* (Tran and Webster, 2011) and their anti-biofilm capacity has been demonstrated against *S. aureus, Pseudomonas aeruginosa*, and *Proteus mirabilis* (Shakibaie et al., 2014). However, to date, the potential of Se nanoparticles against *Mtb* has not been evaluated.

In this study, the mycobactericidal capacity of SeNPs has been demonstrated against two types of mycobacteria, *Msm* and *Mtb*, with MIC values of 0.400 μg/mL and 0.195 μg/mL, respectively. Furthermore, to rule out an effect of the chitosan in the Ch-SeNPs bactericidal action, SeNPs stabilized with a protein such as bovine serum albumin (BSA-SeNPs) have been evaluated on *Mtb* showing an inhibition on the bacterial growth at similar concentrations than Ch-SeNPs (Figure 2A). Therefore, the mycobactericidal effect is due to the SeNPs themselves and not to the presence of the stabilizing agent chitosan. In agreement with the previously demonstrated bactericidal effect of SeNPs on unrelated bacterial pathogens, we have demonstrated that these NPs can also kill Mycobacteria. Furthermore, our analyses by TEM and cryo-EM have shown that SeNPs come into contact with the cell wall of *Mtb* and *Msm*, compromising their integrity and inducing extrusion of cytoplasmic material. These results open a new opportunity for the development of novel nanosystems with high antimycobacterial potential that, alone or in combination with antibiotics, could improve the treatment of multi-drug resistant TB strains.

## Data Availability Statement

The datasets generated for this study are available on request to the corresponding authors.

## Author Contributions

JL-G, RP-R, and BG designed the study, supervised the experimental work, analyzed and interpreted the data, and wrote the manuscript together with HE, AP, JA, and MV-R. HE, AP, and DG performed the experimental work.

## Conflict of Interest

The authors declare that the research was conducted in the absence of any commercial or financial relationships that could be construed as a potential conflict of interest.
