# Supplementary material for: Antimycobacterial Effect of Selenium Nanoparticles on Mycobacterium tuberculosis
Source: Front Microbiol. 2020 Apr 28;11:800. doi: 10.3389/fmicb.2020.00800 (PMC7212347; doi:10.3389/fmicb.2020.00800)
Supplement: Supplementary file 1 [file Table_1.DOCX]

**Supplementary Material**

**
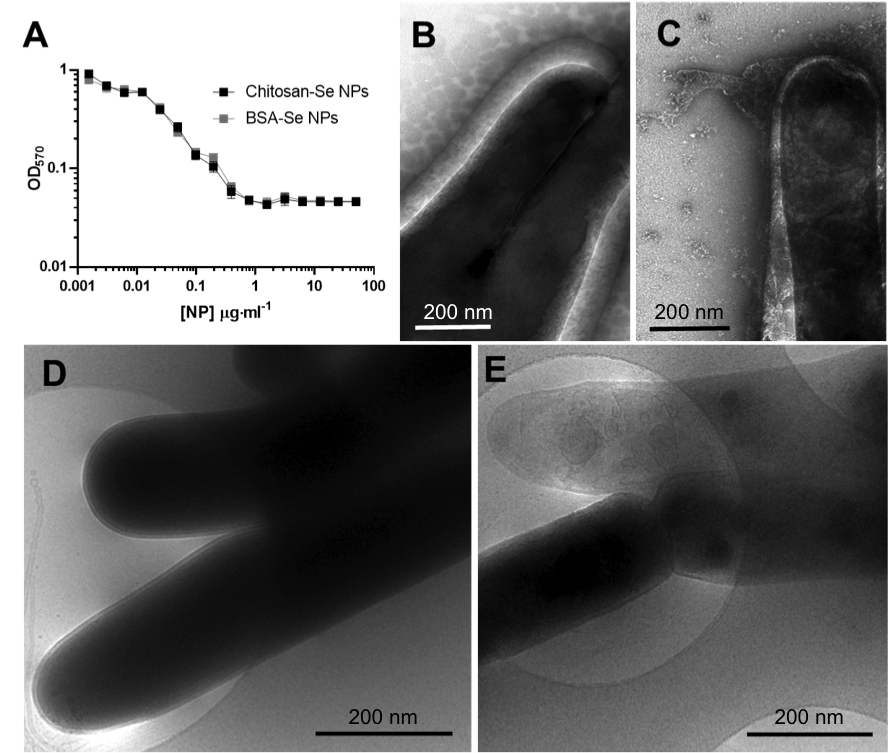
**

**Supplementary Fig. S1.** Antimicrobial activity of Ch-SeNPs and BSA-SeNPs against *Msm*. A) MIC assay testing the antibacterial activity of SeNPs after 7 days of exposure. B-E) TEM and cryo-EM micrographs of *Msm* exposed to 0.4 mg L^-1^ of Ch-SeNPs. B) Control negative staining. C) Ch-SeNPs exposed negative staining. D) Control cryo-EM. E) Ch-SeNPs exposed cryo-EM.

**
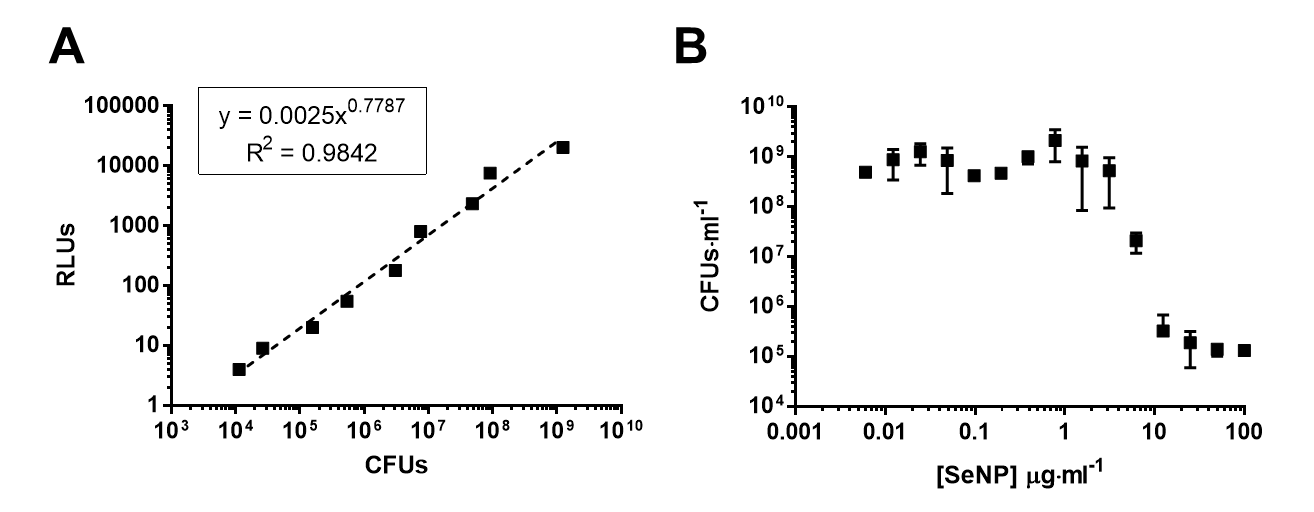
**

**Supplementary Fig. S2.** Antimicrobial activity of SeNPs against *Msm,* measured as luciferase activity. A) Correlation curve between Relative Luminiscence Units (RLUs) and CFUs, showing a linear relationship. B) MIC assay testing the antibacterial activity of SeNPs after 48 h of treatment. The luminescence was measured with an integration time of 10 s.
